# Hydrophobic/Oleophilic Structures Based on MacroPorous Silicon: Effect of Topography and Fluoroalkyl Silane Functionalization on Wettability

**DOI:** 10.3390/nano11030670

**Published:** 2021-03-09

**Authors:** Pilar Formentín, Lluís F. Marsal

**Affiliations:** Departament d’Enginyeria Electrònica, Elèctrica i Automàtica, Universitat Rovira i Virgili, Avinguda Països Catalans, 26 43007 Tarragona, Spain; pilar.formentin@urv.cat

**Keywords:** macroporous silicon, perfluoro-silane, wettability, hydrophobic, oleophilic, contact angle

## Abstract

The effect of the morphology and chemical composition of a surface on the wettability of porous silicon structures is analyzed in the present work. Hydrophobic and superhydrophobic macroporous substrates are attractive for different potential applications. Herein, different hydrophobic macroporous silicon structures were fabricated by the chemical etching of p-type silicon wafers in a solution based on hydrofluoric acid and coated with a fluoro silane self-assembled monolayer. The surface morphology of the final substrate was characterized using a scanning electron microscope. The wettability was assessed from contact angle measurements using water and organic solvents that present low surface energy. The experimental data were compared with the classical wetting states theoretical models described in the literature. Perfluoro-silane functionalized macroporous silicon surfaces presented systematically higher contact angles than untreated silicon substrates. The influence of porosity on the surface wettability of macoporous silicon structures has been established. These results suggest that the combination of etching conditions with a surface chemistry modification could lead to hydrophobic/oleophilic or superhydrophobic/oleophobic structures.

## 1. Introduction

The wettability of solid surfaces plays important roles not only in industrial applications [1,2,3] but also in daily life [4,5,6]. It is a characteristic property of materials and it is determined by two factors: one is the chemical component of the surface and the other is the surface topology [7,8,9,10,11,12,13,14]. Superhydrophobic surfaces (SHSs) with a water contact angle (WCA) larger than 150° have attracted interest due to developments in nanotechnology and their applications in industry [15,16,17,18,19]. This type of surface can be prepared by a combination of lowering the surface energy and increasing the surface roughness [20]. Natural SHSs have been observed in some plant leaves and insect wings. Several studies have been focused towards the fabrication of synthetic superhydrophobic surfaces capable of mimicking the natural SHSs [21,22,23,24,25,26]. A promising method to produce silicon superhydrophobic surfaces is the use of porous silicon.

Macroporous silicon (MacroPSi) is a controllable material in respect of its pore size distributions depending on its fabrication conditions [27,28,29]. The wetting properties of porous silicon depend on pore diameter and porosity. Nanoporous silicon is reported to be hydrophilic, while macroporous silicon can be either hydrophilic or highly hydrophobic, depending on the silicon doping and the electrochemical etching conditions [30,31]. The self-assembly of the alkylsilane or fluoride silane are some of the methods to introduce low surface energy compounds on the surface to enhance the hydrophobicity [12,31,32,33]. Perfluoro-silane modified materials present improve dielectric properties and increase the stability of some devices, such as organic solar cells or transistors [34,35,36].

In this work, we present the fabrication and characterization of MacroPSi surfaces and how the wettability of the surface depends on the porosity and the surface tension of the liquid used on the contact angle measurements. We demonstrate that the water repellence of the MacroPSi samples is subject to the resistivity of the silicon wafer and the etching parameters, and the non-wetting properties can be enhanced by surface fluorocarbon modification.

Considering the biocompatibility of porous silicon, such surfaces will be useful to the silicon-based biosensors, microarrays or microfluidics.

## 2. Materials and Methods

### 2.1. Fabrication of MacroPSi Structures

Boron-doped silicon wafers of *p* < 100 > crystal orientation and different resistivity were used for the experiments (Si-Mat, Germany). MacroPSi substrates were prepared in a custom-made Teflon etching cell using an electrolyte of hydrofluoric acid (HF 48%, Sigma-Aldrich, Germany) in N, N dimethylformamide (DMF, Sigma-Aldrich, Germany) (1:3). The anodization process was carried out during 90 min, changing the current density, 10 or 20 mA/cm^2^, and the resistivity of the silicon substrate. Then, the samples were rinsed with pentane and dried under a nitrogen flow.

### 2.2. Surface Characterization

MacroPSi samples were morphologically characterized by environmental scanning electron microscopy (ESEM) using an FEI Quanta 600 environmental scanning electron microscope (Hillsboro, OR, USA) operating at an accelerating voltage of 25 keV.

### 2.3. Surface Modification of MacroPSi

Macroporous silicon samples were modified with 1H, 1H, 2H, 2H-Perfluorooctyltrichloro silane (FOTS, CAS: 78560-45-9, Alfa Aesar, Germany) via physical vapor deposition. The substrates were place in a sealed vessel with a container filled with silane precursor liquid. There was no direct contact between the liquid and the substrates. The vessel was 1 h under low pressure at room temperature followed by annealing at 120 °C for 2 h.

### 2.4. Contact Angle Measurements

Contact angles (CAs) were measured using a drop shape analysis system under ambient conditions applying solvent droplets of approximately 3 µL. The average contact angle value was obtained by measuring 9 times at 3 different positions for each sample.

## 3. Results and Discussions

### 3.1. Fabrication and Characterization of MacroPSi Substrates

The MacroPSi substrates were fabricated by electrochemical etching of p-type Si wafers via anodization [37,38,39,40]. The resistivity of the silicon wafer and the current density during the etching process were varied in order to generate porous surfaces with two different porosities. The surface morphology of macroporus silicon was characterized by ESEM and their wettability was measured on an optical contact angle meter.

**Figure 1:**
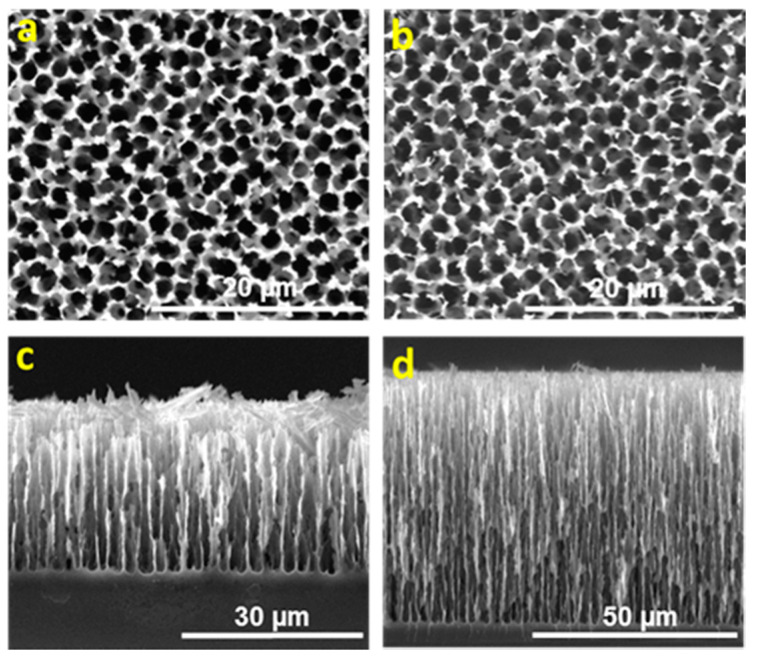
Environmental scanning electron microscopy (ESEM) image of a top and cross-sectional view of macroporous silicon (MacroPSi) substrates with 6.2 Ωcm resistivity: (**a**,**c**) MacroPSi-1: HF:DMF = 1:3, current density = 10 mA/cm^2^, etching time = 90 min and porosity = 74.9 ± 6.5%; (**b**,**d**) MacroPSi-2: HF:DMF = 1:3, current density = 20 mA/cm^2^, etching time = 90 min and porosity = 86.2 ± 6.2%.

**Figure 2:**
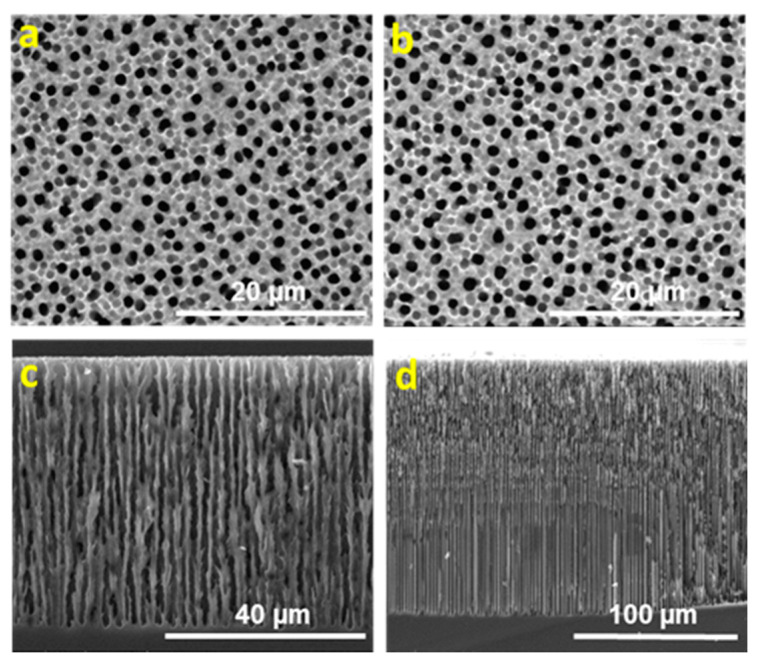
ESEM image of top and cross-sectional view of MacroPSi substrates with 10 Ω cm resistivity: (**a**,**c**) MacroPSi-3: HF:DMF = 1:3, current density = 10 mA/cm^2^, etching time = 90 min and porosity = 30.2 ± 1.4%; (**b**,**d**) MacroPSi-4: HF:DMF = 1:3, current density = 20 mA/cm^2^, etching time = 90 min and porosity = 27.5 ± 0.4%.

ESEM images have been analyzed using ImageJ software. Silicon substrates with a pore size of between 1.3 and 1.8 µm were obtained. The porosity value has been computed from the surface topography by counting the percentage of pixels whose intensity is below a set threshold. The measured porosity is the “surface porosity” of the sample, that is, the area fraction occupied by depressions. Figure 1 and Figure 2 show the scanning electron micrographs of the pore morphologies and cross view for each of the four studied surfaces.

The influence of the resistivity of the substrate on the pore density and pore depth is similar to the previous studies [41]. If the HF:DMF ratio, current density and etching time remain constant, pore density decreases with the increasing of substrate resistivity (Figure 1a,b and Figure 2a,b). As shown in Figure 1c,d and Figure 2c,d, the pore length increases with the increasing of substrate resistivity. Table 1 summarizes the porosity and the pore depth obtained under different experimental conditions.

### 3.2. Hydrophobic and Superhydrophobic States on MacroPSi

**Figure 3:**
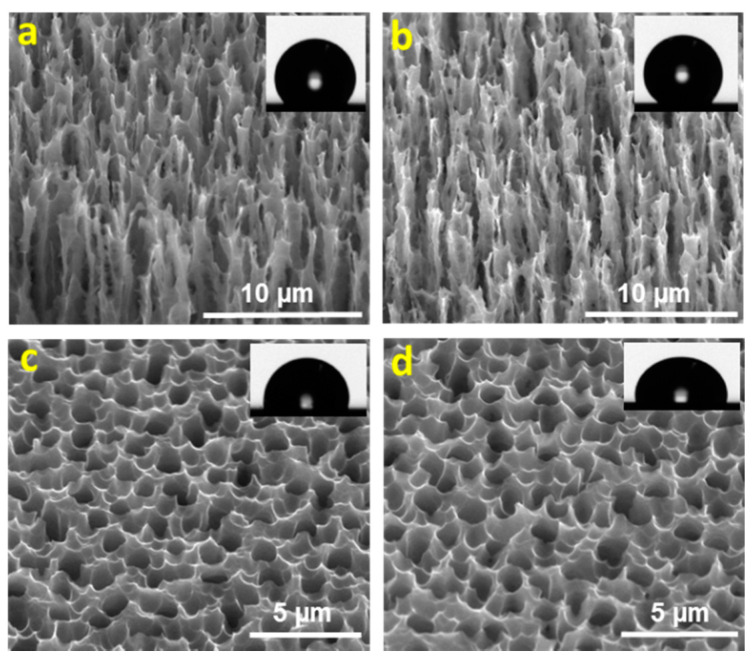
ESEM images of the studied macroporous silicon substrates: (**a**) MacroPSi-1; (**b**) MacroPSi-2; (**c**) MacroPSi-3; (**d**) MacroPSi-4.

MacroPSi surfaces were prepared on silicon wafers tuning the anodization conditions in order to display their hydrophobic behavior. The reason for the changed wettability is due to the obtained morphology, which depends on the porosity of the final substrate. To have repellence, surfaces with more micropores are favorable because of the greater air resistance against wettability. In Table 2, we show the contact angle (CA) measurements for each of the four studied surfaces. In this work, the contact angles were calculated directly by measuring the angle formed between the solid and the tangent to the drop surface. The substrates were analyzed after fabrication and after more than 4 months at ambient conditions. We observed that the hydrophobicity of the samples increases with the increasing of the porosity. This effect could be explained by the increasing level of porous silicon fractalization and the topography, which reduce the contact of the solid surface with the drop of the liquid [31] (Figure 3).

**Figure 4:**
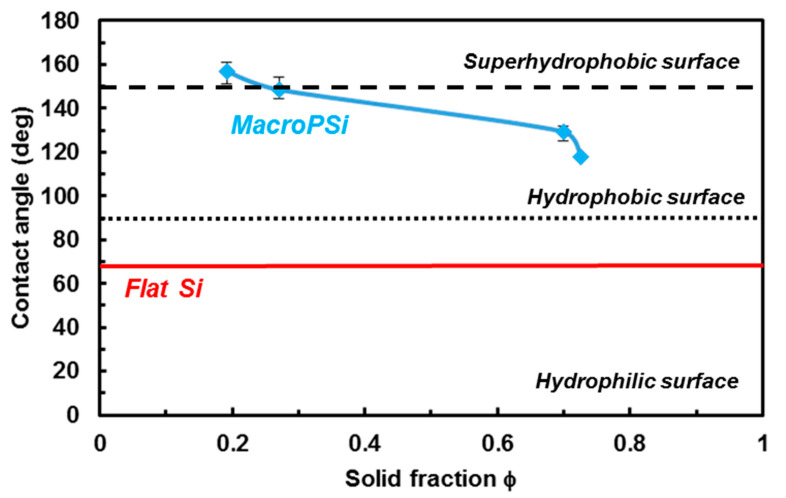
Experimental water contact angle results of MacroPSi substrates as a function of solid fraction.

Figure 4 illustrates the water contact angles (WCAs) on porous structures as a function of solid fraction (Φ), which was calculated by 1-Apore/A, where Apore is the summation of the pore area in a given area (A) [42]. It was measured several times in order to check the uniformities of the surface. The results show the influence of the solid fraction on the wettability of the structure. On MacroPSi substrates, the CA changed from 117.9° to 157.0° with a solid fraction from 0.7 to 0.2. In the case of flat silicon (Φ = 1), the CA was 69°.

Another parameter to consider is the oxidation of the substrates. The porosity could also be the cause of that and then the reduction of the water contact angle on the porous substrates measured several months after fabrication under ambient conditions. This effect is higher on MacroPSi-1 and MacroPSi-2 with porosities superior to 70% (Table 2), which are more hydrophobic, and then the oxidation is higher than in substrates with less porosity.

**Figure 5:**
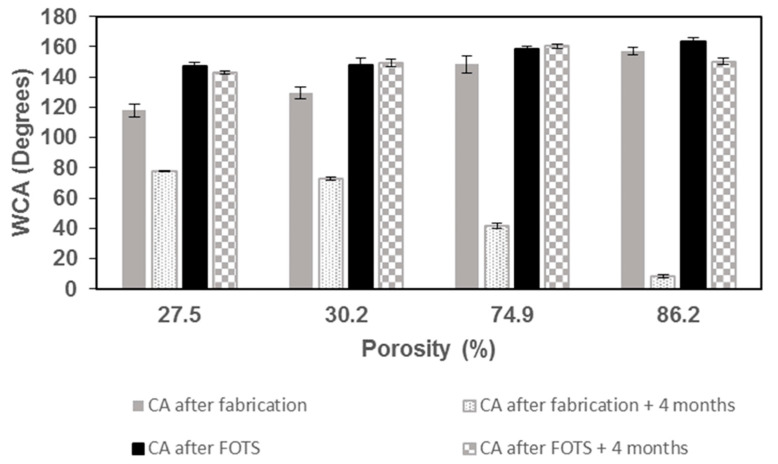
Water contact angle (WCA) as a function of porosity: values of WCA for MacroPSi surfaces after fabrication, after 4 months of fabrication, after FOTS modification and after 4 months of FOTS functionalization.

Surface functionalization with hydrophobic termini improves the water repellence of the porous substrates. We modified the substrates by a chemical treatment using hydrophobic surface coupling agent. MacroPSi substrates showed an increase in CA values after modification with FOTS (Figure 5). These values are also affected by the porosity. We observed that the contact angle increases with the degree of porosity and after the functionalization, which generate superhydrophobic states on MacroPSi-1 and MacroPSi-2, increasing CA values from 148.4 ± 5.8° to 158.5 ± 2.1° and from 157.0 ± 2.5° to 163.2 ± 2.9°, respectively. The obtained structures were stable and exhibited no decay in CA value over 4 months; just MacroPSi-2 demonstrated oxidation of the surface, causing a reduction in CA value after these months. As we have commented previously, the oxidation is related to the high porosity of the surface. These results indicate that the surface modification with a fluoro-silane protects against the oxidation, and therefore the variation of the substrate wettability is smaller than without functionalization after several months.

### 3.3. Intermediate Wetting State on MacroPSi Structures

In order to understand how the interfacial properties affect the introduction of molecules into the pores of macro- or nanoporous materials, it is important to improve studies of liquid–solid interactions. The contact angle (*θ*) is used as a measure of wetting between a liquid and a solid surface. On a smooth surface, this value is given by Young’s equation [43], where γsv, γsl and γlv are the interfacial tensions of the solid–vapor, solid–liquid and liquid–vapor interface (Equation (1)).

γlv cos *θY* = γsv − γsl (1)

**Figure 6:**
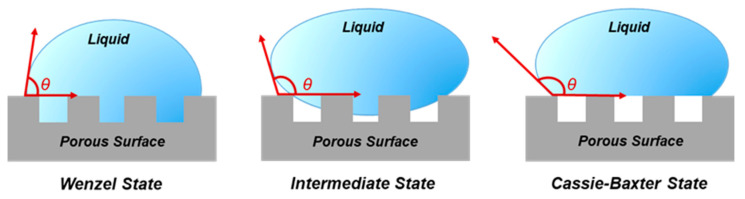
Schematic representation of the wetting states of a liquid droplet on non-planar surfaces.

Wenzel [44] studied contact angle on rough surfaces, where the liquid may completely penetrate into the rough structure and the CA on the surface is given by Equation (2). In this equation, r is the area ratio of structured surface to planar surface. Cassie and Baxter expanded upon Wenzel’s model to allow for measurements of porous surfaces (Equation (3)). In this case, the droplet of the liquid does not entirely cover the rough surface and leaves air between the droplet and the substrate [45,46,47],

cos *θ_w_* = *r* cos *θ_Y_* (2)

cos *θ_CB_* = (cos *θ_Y_* + 1) *f* − 1 (3)

where the contact angle depends on the surface fraction (*f*) of the droplet that is in contact with the surface. It was introduced as a factor for the partial wetting model and represents the part of liquid wetting into the structures. It is clear that f ranges from *f* = 0 for the non-wetted Cassie–Baxter state to *f* = 1 for the fully wetted Wenzel state (Figure 6). However, a perfect state model is infrequent in real experiments. An intermediate state occurs between the Wenzel and Cassie–Baxter states, and several experimental water CAs disagree with these models [42].

**Figure 7:**
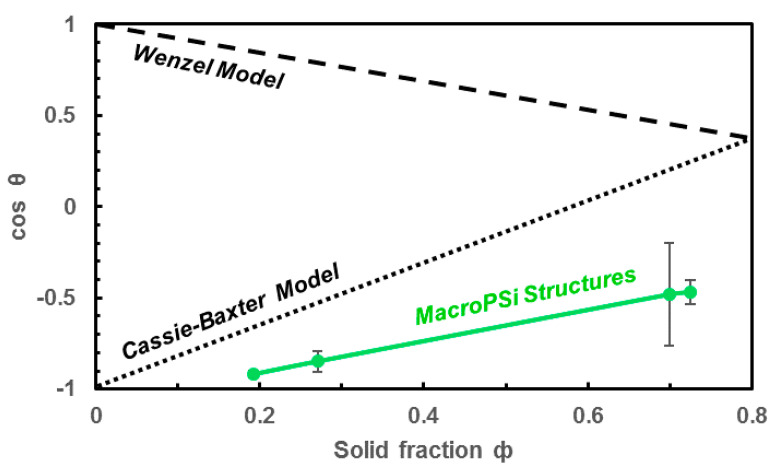
Comparison of experimental data of WCAs at MacroPSi surfaces and theoretical models.

Figure 7 illustrates a comparison between contact angles at MacroPSi substrates and the Wenzel and Cassie–Baxter theoretical models as is reported in Reference [42]. In this published work, a theoretical partial wetting model is developed in order to explain that in structured surfaces there is a derivation between experimental and theoretical models for hydrophobic substrates. We have not used the equations presented in the literature, but, if we compare the published graphics with our results (Figure 7), it shows better agreement with the model established by Nagayama et al. [42] than with the classic models. It means that the experimental WCAs for MacroPSi substrates with a porosity ranging from 27.5% to 86.2% show agreement with a partial wetting model.

### 3.4. Wetting Behavior of Different Liquids on Macroporous Structures

Most studies on macroporous silicon have been focused on measurements of water contact angle. Herein, we have studied the wetting behaviour of liquids with different viscosities and low surface tension on macroporous silicon surfaces. Table 3 shows the parameters of the liquids at room temperature used in this work. Contact angle (CA) measurements for MacroPSi substrates with different porosities using four different liquids are summarized in Table 4. The aim of this study is to know the effect of surface morphology and the porosity on the wettability of the structure with different solvents. We note that flat silicon presents a contact angle of 69° using deionized (DI) water, and only contact angles of macroporous silicon substrates are reported in this table.

**Figure 8:**
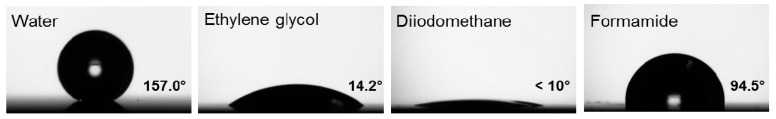
Images of a liquid droplet on MacroPSi-2 with a porosity of 86.2 ± 6.2%.

MacroPSi structures not only show water repellency but are also an excellent absorber for organic solvents. We observed higher CA values using water as a solvent and lower CA values with a droplet of ethlynene glycol, diiodomethane or formamide. As a result, we obtained macroporous silicon hydrophobic/oleophilic surfaces (Figure 8).

Wettability is also related to the topography. When we compare the same liquid but with a different surface morphology, the CA depends on the porosity of the substrate. In the case of the DI water, the hydrophobicity is higher when the porosity increases. When using ethylene glycol or diiodomethane, the effect of the porosity is the opposite. The CA is higher when the porosity decreases. In the case of formamide, the effect of the porosity is not so remarkable in the CA values.

After chemical modification with FOTS, all the contact angle values increased, as we expected. The wettability of the surface changes from hydrophobic to superhydrophobic for water and from olehophilic to oleophobic for the probe liquids with low surface tension. In this case, the topography is not decisive. If we compare samples with different porosities, the CA value increases slightly higher when the porosity increases, but it is not a significant value (Table 5).

**Figure 9:**
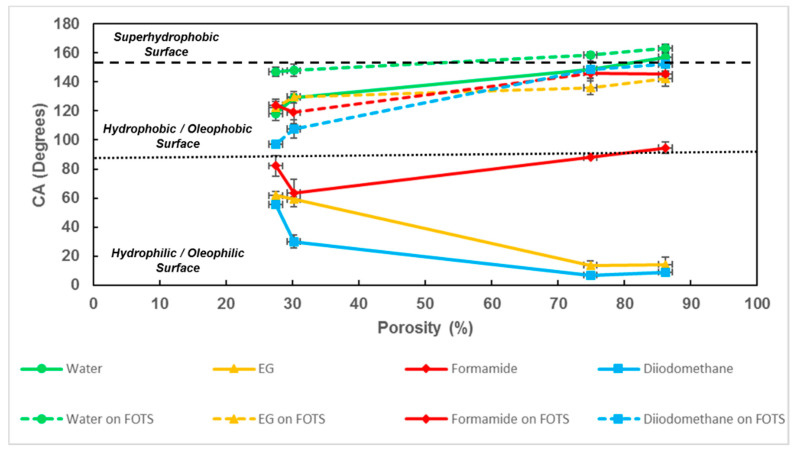
Comparison of CAs for different solvents at MacroPSi and MacroPSi-FOTS versus porosity.

Figure 9 illustrates the experimental CA values of the studied solvents on MacroPSi and MacroPSi-FOTS structures. The results obtained from this graphic indicate which wetting state presents the porous surface. For organic solvents, such as diiodomethane, ethylene glycol and formamide, the macroporous structure fabricated is oleophilic and proves hydrophobic if the liquid is water. As is shown in the figure, the CA value increases with porosity for water or formamide, but it decreases when the solvent studied is ethylene glycol or diiodomethane. The reason for this could be the surface tension of these liquids, which is lower than the value for water or formamide. It seems like the porosity increases this effect and the surface gets more hydrophilic. After modification with FOTS, the CA values with organic solvents increase and then the surface becomes oleophobic. In the case of water, after surface modification, the porous surface changes from hydrophobic to superhydrophobic for porosities higher than 70%.

## 4. Conclusions

The present work demonstrates macroporous silicon processing as a method to obtain surfaces with controlled wetting behaviour. Hydrophobic/oleophilic and superhydrophobic/oleophobic surfaces on MacroPSi substrates were prepared by electrochemical etching combining with chemical modification with a hydrophobic coupling agent by chemical vapour deposition. The liquid–solid interfaces were studied with the contact angle measurements. The results show that the wetting behaviour depends on the substrate morphology. The contact angle increases with an increasing of the porosity, and surface functionalization with hydrophobic termini improves the water repellence of the porous substrates.

We have also analyzed the wetting behaviour on MacroPSi surfaces of liquids with different surface tensions. The contact angle value decreases using liquids with low surface tension, such as ethylene glycol. If the surface is previously functionalized, no significant differences are observed if we compare the CA values with water and organic solvents, but it should be noted that in the case of water we obtained a superhydrophobic surface for porosities higher than 70%. These results could be used to design both hydrophobic/oleophilic and superhydrophobic/oleophobic surfaces, which are of great significance for practical applications in liquid microtransport in microfluid devices and microsystems/labs on a chip.

## Figures and Tables

**Table 1:** Resistivity, anodization current density, resulting porosity and pore depth for macroporous silicon substrates.

Substrates	Resistivity (Ω cm)	Currrent Density (mA/cm^2^)	Porosity (%)	Pore Depth (µm)
MacroPSi-1	6.2	10	74.9 ± 6.5	27.0 ± 0.7
MacroPSi-2	6.2	20	86.2 ± 6.2	57.8 ± 3.9
MacroPSi-3	10	10	30.2 ± 1.4	51.7 ± 3.1
MacroPSi-4	10	20	27.5 ± 0.4	155.8 ± 1.3

**Table 2:** Values of water contact angle in degrees for macroporous silicon substrates.

	MacroPSi-1	MacroPSi-2	MacroPSi3	MacroPSi-4
Porosity (%)	74.9 ± 6.5	86.2 ± 6.2	30.2 ± 1.4	27.5 ± 0.4
CA (°)	148.4 ± 5.8	157.0 ± 2.5	129.3 ± 4.0	117.9 ± 4.3
CA (°)	41.4 ± 1.9	<10	72.9 ± 0.8	77.7 ± 0.2
After +4 months				

**Table 3:** Surface tension and viscosity of liquids employed in this work.

Liquid	Surface Tension (mN/m)	Dynamic Viscosity (mPa s)
DI Water	72.8	1.0
Ethylene glycol	48.0	17.3
Diiodomethane	50.8	2.8
Formamide	58.0	1.0

Note: All data cited from [48,49].

**Table 4:** Summary of contact angle values for MacroPSi substrates using different solvents.

Substrates	Porosity (%)	Water CA (deg)	EthyleneGlycol CA (deg)	Diiodomethane CA (deg)	Formamide CA (deg)
MacroPSi-1	74.9 ± 6.5	148.4 ± 5.8	13.7 ± 2.8	<10	88.0 ± 0.7
MacroPSi-2	86.2 ± 6.2	157.0 ± 2.5	14.2 ± 5.0	<10	94.5 ± 4.0
MacroPSi-3	30.2 ± 1.4	129.3 ± 4.0	59.1 ± 1.8	30.0 ± 4.3	63.5 ± 9.7
MacroPSi-4	27.5 ± 0.4	117.9 ± 4.3	61.6 ± 3.0	55.5 ± 1.0	82.2 ± 7.0

**Table 5:** Summary of contact angle values for MacroPSi substrates after FOTS modification using different solvents.

Substrates	Porosity (%)	Water CA (deg)	Ethylene Glycol CA (deg)	Diiodomethane CA (deg)	Formamide CA (deg)
MacroPSi-1	74.9 ± 6.5	158.5 ± 2.1	136.1 ± 4.7	148.7 ± 8.1	146.2 ± 3.9
MacroPSi-2	86.2 ± 6.2	163.2 ± 2.9	142.4 ± 5.1	152.3 ± 8.6	145.3 ± 1.5
MacroPSi-3	30.2 ± 1.4	148.0 ± 4.2	129.7 ± 2.2	107.7 ± 6.3	119.0 ± 7.8
MacroPSi-4	27.5 ± 0.4	147.0 ± 3.0	122.6 ± 3.9	97.1 ± 1.5	124.1 ± 4.2

## Data Availability

Data are contained within the present article.
